# Supplementary material for: Genome-wide discovery of cis-regulatory elements in a large genome
Source: eLife. 2026 Oct 1;15:RP111378. doi: 10.7554/eLife.111378 (PMC13630387; doi:10.7554/eLife.111378)
Supplement: Peer review history

# Editor report: eLife Assessment

**DOI:** 10.7554/eLife.111378.3.sa0

**Authors:** Kristin Tessmar-Raible

**Affiliations:** University of Vienna Austria

**Keywords:** Compelling, Important

This **important** study combines chromatin accessibility and genomic DNA sequence conservation data from low-coverage genome sequencing of related species (without assembly), for the in silico identification of *cis*-regulatory elements in large genomes. The approach and results are **compelling** and well supported by the experimental validations. The work will be of interest to researchers working in the field of gene regulation and evolution, particularly because the methodology proposed can be applied to a large variety of experimental organisms.

# Referee report: Reviewer #1 (Public review)

**DOI:** 10.7554/eLife.111378.3.sa1

Summary:

Forbes et al. developed an integrated approach to identify cis-regulatory elements (CREs) in the large (3.6 Gbp) genome of the crustacean Parhyale hawaiensis, addressing the challenge of pinpointing these regions among large regions of non-coding sequences. They combined ATAC-seq chromatin accessibility profiling (both bulk and single-nucleus) across embryonic and adult tissues with low-coverage genome sequencing of three congeneric species (P. aquilina, P. darvishi, P. plumicornis). Without assembling congener genomes, they mapped reads with low stringency to the P. hawaiensis reference, identifying about 55k conserved islands that overlap ATAC peaks more than expected by chance. This dual filter was used to select CRE candidates for transgenic reporter validation, yielding 6 functional elements (out of 11 tested) driving ubiquitous, neuronal, or muscle-specific expression, a major advance for non-model systems with large genomes.

Strengths:

Forbes et al. generated high-quality ATAC data across multiple scales. Using bulk ATAC-seq (from whole embryos, developing and adult legs) they identified tens of thousands of open chromatin peaks across the assembled P. hawaiensis large genome. Moreover, using single-nucleus ATAC-seq from adult legs, they could resolve differentially accessible chromatin profiles across more than 15 cell types previously identified by scRNA-seq, enabling cell-type-specific candidate selection.

Furthermore, their innovative low-coverage comparative genomics method mapped 0.46-6.4% of congener reads to P. hawaiensis without genome assembly, revealing hundreds of thousands of conserved non-coding islands, including about 55k showing conservation in all four species, far exceeding random expectation.

Using the developed approach, the authors could validate 6 (out of 11 candidates) reporter constructs, driving robust ubiquitous and tissue-specific expression, succeeding where prior promoter-only screening failed and providing immediately useful genetic tools for the Parhyale community.

Weaknesses:

The primary limitation is that functional CRE testing was performed only in P. hawaiensis. While the conservation maps provide a valuable resource for comparative analyses, functional validation in congener species was not performed, so the extent to which the identified CREs or the prioritization strategy can be functionally generalized across related species remains to be established.

The approach did not successfully identify developmental CREs among the candidates tested. None of the candidates selected using the combined ATAC-seq and conservation filtering drove reporter expression matching the expected endogenous patterns. The authors appropriately discuss possible technical and biological explanations.

Overall Assessment:

Forbes et al. fully succeed with their integrated approach to (1) generate an ATAC-seq atlas plus functional CRE discovery and (2) innovative low-coverage sequencing for conservation mapping in the large 3.6 Gbp genome of Parhyale hawaiensis. Their combination of ATAC-seq chromatin accessibility profiling (bulk and single-nucleus) across embryonic and adult tissues with low-coverage genome sequencing of three congeneric species (P. aquilina, P. darvishi, P. plumicornis), without congener genome assembly, drastically shrank the CRE search space. Using this approach, the authors could validate six out of 11 candidate transgenic reporters (ubiquitous, neuronal, and muscle-specific) where prior promoter-only screening failed.

The low-coverage mapping innovation cuts cost and labour while snATAC-seq provides cell-type resolution, making these resources valuable for building new genetic and imaging tools in Parhyale.

This compelling method also has the potential to enable labs with limited resources to identify and characterize regulatory elements in more non-model organisms, advancing our understanding of their evolution while establishing a scalable pipeline for large-genome systems.

Comments on revised version.

The authors have adequately addressed all my previous comments. I have no further specific suggestions or requests.

# Referee report: Reviewer #2 (Public review)

**DOI:** 10.7554/eLife.111378.3.sa2

The manuscript by Forbes, Skafida, Karapidaki et al. concerns the in-silico identification of cis-regulatory elements (CREs) in large genomes using chromatin accessibility (ATAC-seq) and sequence conservation (genomic DNA sequencing) data. They exemplify this method by applying it to identify novel CREs in Parhyale hawaiensis, which they validated using reporter constructs.

The results are convincing and are well supported by the data and validations. Identified CREs are valuable for researchers interested in the regulation of the expression of genes they control.

The methodology on the whole is also valid, as suggested by the results and previous publications on various taxa. Sequence conservation, as stated by the authors, was long used as a method to identify regions of non-coding DNA with functional and evolutionary constraints. The same applies to ATAC-seq data, which has also been used as a proxy for functional regions in different animals such as sea urchins and amphioxus. The methodology proposed is likely to be successfully used by researchers working on a variety of experimental organisms.

The authors do not use existing genome assemblies and use short-read sequencing to identify conserved regions, and while it is not conceptually novel, such an approach is becoming more and more viable and useful considering the recent advances in next generation sequencing technology and the decrease in price of short-read sequencing.

The authors have addressed and discussed the limitations and weaknesses of the approach as well as explicitly indicated the advantages.

All in all, the authors provide a valid method to strengthen CRE identification via sequence conservation without the need of multiple complete close species genome assemblies, making it a compelling option for non-model organism research.

# Referee report: Reviewer #3 (Public review)

**DOI:** 10.7554/eLife.111378.3.sa3

Summary:

Forbes et al. present a new approach for identifying cis-regulatory elements in large genomes. Using Parhyale hawaiensis, a crustacean with a large genome (~3.6 Gb, comparable in size to the human genome), the authors show that current methods for identifying cis-regulatory elements, effective in smaller genomes, are markedly inefficient in organisms with large genomes. To address this limitation, they combine bulk ATAC-seq and single-cell (sc) ATAC-seq to identify chromatin regions that are either ubiquitously accessible or specifically accessible in particular cell types. They further integrate comparative genomics across multiple Parhyale species (P. hawaiensis, P. aquilina, and P. darvishi), selected at appropriate phylogenetic distances (20-95 million years divergence), to pinpoint conserved open chromatin regions likely under functional constraint.

Using this strategy, the authors predict a set of ubiquitous and cell-type-specific cis-regulatory elements. Importantly, they validate these predictions using rigorous transgenic reporter assays, convincingly demonstrating that their approach can successfully identify functional regulatory elements where previous methods had failed.

Strengths:

The approach introduced by Forbes et al. is conceptually straightforward, efficient, and readily transferable to other organisms. The validation experiments show not only that a substantial proportion of the predicted elements are functional, but also that the method is capable of identifying both ubiquitous and cell-type-specific regulatory elements. Given that the identification of regulatory regions remains a major bottleneck in understanding the molecular mechanisms underlying processes of development and regeneration, this work has the potential to make a significant impact in developmental and regeneration biology, particularly for studies involving non-model organisms with large genomes.

An additional strength is the demonstration that only the genome of the focal species requires high-quality sequencing and assembly. In contrast, species used solely for comparative analysis can be sequenced at low coverage without assembly, substantially reducing costs and increasing the accessibility of the approach.

Weaknesses:

While the method is effective in identifying regulatory elements that are active ubiquitously or in differentiated cell types, it failed in detecting elements associated with developmentally regulated genes. This may be due to trivial reasons, such as very low level of expression of the selected genes. However, as acknowledged by the authors, it may also indicate inherent challenges in identifying regulatory elements associated with developmentally dynamic gene regulation, compared to those associated with genes expressed in differentiated cell types.

A second limitation, also acknowledged by the authors, is the absence of chromatin conformation capture data, which would help link distal regulatory elements to their target genes. This limitation may be particularly relevant for developmentally regulated genes, where long-range regulatory interactions may be critical.

Addressing these limitations will be an important direction for future work. Nonetheless, the approach as presented in this manuscript represents a key contribution that sets the stage for further methodological advances in the identification of cis-regulatory elements in large genomes.

Comments on revised version.

I am fully satisfied with the current version of the manuscript.

# Author comment: Author response

**DOI:** 10.7554/eLife.111378.3.sa4

**Authors:** Gillian Forbes, Emilia Skafida, Irene Karapidaki, Savannah Moinet, Mowgli Dandamudi, Çağrı Çevrim, Farzaneh Momtazi, Chryssa Anastasiadou, Sabrina Lo Brutto, Michalis Averof, Mathilde Paris

**Affiliations:** Institut de Genomique Fonctionnelle de Lyon (IGFL) – CNRS Lyon France; Institut de Genomique Fonctionnelle de Lyon (IGFL) – CNRS Lyon France; Institut de Genomique Fonctionnelle de Lyon (IGFL) – CNRS Lyon France; Institut de Genomique Fonctionnelle de Lyon (IGFL) – CNRS Lyon France; Institut de Genomique Fonctionnelle de Lyon (IGFL) – CNRS Lyon France; Institut de Genomique Fonctionnelle de Lyon (IGFL) – CNRS Lyon France; Senckenberg Research Institute and Natural History Museum Frankfurt Germany; Fisheries Research Institute Nea Peramos Greece; University of Palermo Palermo Italy; Institut de Genomique Fonctionnelle de Lyon (IGFL) – CNRS Lyon France; Institut de Genomique Fonctionnelle de Lyon (IGFL) – CNRS Lyon France

The following is the authors’ response to the original reviews.

**Reviewer #1 (Public review):**

Summary:

Forbes et al. developed an integrated approach to identify cis-regulatory elements (CREs) in the large (3.6 Gbp) genome of the crustacean Parhyale hawaiensis, addressing the challenge of pinpointing these regions among large regions of non-coding sequences. They combined ATAC-seq chromatin accessibility profiling (both bulk and single-nucleus) across embryonic and adult tissues with low-coverage genome sequencing of three congeneric species (P. aquilina, P. darvishi, P. plumicornis). Without assembling congener genomes, they mapped reads with low stringency to the P. hawaiensis reference, identifying about 55k conserved islands that overlap ATAC peaks more than expected by chance. This dual filter was used to select CRE candidates for transgenic reporter validation, yielding 6 functional elements (out of 11 tested) driving ubiquitous, neuronal, or muscle-specific expression, a major advance for non-model systems with large genomes.

Strengths:

Forbes et al. generated high-quality ATAC data across multiple scales. Using bulk ATAC-seq (from whole embryos, developing and adult legs), they identified tens of thousands of open chromatin peaks across the assembled P. hawaiensis large genome. Moreover, using single-nucleus ATAC-seq from adult legs, they could resolve differentially accessible chromatin profiles across over 15 cell types previously identified by scRNA-seq, enabling cell-type-specific candidate selection.

Furthermore, their innovative low-coverage comparative genomics method mapped 0.46-6.4% of congener reads to P. hawaiensis without genome assembly, revealing hundreds of thousands of conserved non-coding islands, including about 55k showing conservation in all four species, far exceeding random expectation.

Using the developed approach, the authors could validate 6 (out of 11 candidates) reporter constructs, driving robust ubiquitous and tissue-specific expression, succeeding where prior promoter-only screening failed and providing immediately useful genetic tools for the Parhyale community.

Weaknesses:

The primary limitation is that functional CRE testing was performed only in P. hawaiensis. While conservation maps are valuable resources, the manuscript lacks functional validation in congener species, limiting claims about broad applicability across related genomes/species.

The approach also failed to validate developmental CREs. None of the candidates from combined ATAC and conservation filtering drove reporter expression matching endogenous patterns. The authors appropriately hypothesize technical limits (low expression) or biological factors (long-range enhancers, shadow enhancers).

Overall Assessment:

Forbes et al. fully succeed with their integrated approach to (1) generate an ATAC-seq atlas plus functional CRE discovery and (2) innovative low-coverage sequencing for conservation mapping in the large 3.6 Gbp genome of Parhyale hawaiensis. Their combination of ATAC-seq chromatin accessibility profiling (bulk and single-nucleus) across embryonic and adult tissues with low-coverage genome sequencing of three congeneric species (P. aquilina, P. darvishi, P. plumicornis), without congener genome assembly, drastically shrank the CRE search space. Using this approach, the authors could validate six out of 11 candidate transgenic reporters (ubiquitous, neuronal, and muscle-specific), where prior promoter-only screening failed.

The low-coverage mapping innovation cuts cost and labour while snATAC-seq provides cell-type resolution, making these resources valuable for building new genetic and imaging tools in Parhyale.

This compelling method also has the potential to enable labs with limited resources to identify and characterize regulatory elements in more non-model organisms, advancing our understanding of their evolution while establishing a scalable pipeline for large-genome systems.

We thank the reviewer for their comments and valuable feedback.

**Reviewer #1 (Recommendations for the authors):**

(1) Standardize terminology and introduce acronyms properly:

I suggest standardizing technique names throughout the manuscript (e.g., ATAC-seq rather than ATACseq, RNA-seq rather than RNAseq, ChIP-seq rather than ChipSeq). Please also introduce technical terms with their full name at first use, such as 'Assay for Transposase-Accessible Chromatin using sequencing (ATAC-seq)' rather than just the acronym.

Corrected.

(2) Correct author name:

On page 15, "Lo brutto" appears to be misspelt. Please review and fix throughout the text and the corresponding reference in the bibliography if this is the case.

Corrected.

(3) Revise the title to reflect dual contributions:

The paper delivers two key advances: (a) ATAC-seq atlas plus functional CRE discovery in P. hawaiensis, and (b) innovative low-coverage sequencing for conservation mapping across congenerics. Current title highlights only the first. Consider modifying the title to better reflect both.

The title reflects our overall objective, without highlighting one of the two approaches (advances) in particular. We would like to keep this concise title and invite readers to read about the two approaches in the abstract.

(4) Emphasize the combined power of the approach in the Discussion and Conclusions:

A significant innovation of this manuscript is integrating low-coverage comparative genomics with ATAC-seq to prioritize functional CREs in a large non-model genome. The abstract highlights this well, but the Discussion and Conclusions could better emphasize the power of this pipeline over ATAC-seq alone. The authors could also add 2-3 sentences quantifying cost savings versus traditional assemblies and reiterating how this complements ATAC-seq for efficient CRE prioritization in non-model species.

We have added the following text in the Discussion to describe complementary contributions of ATAC-seq and sequence conservation to CRE discovery: "Previous efforts to identify cis-regulatory elements in Parhyale relied on reporter constructs carrying a few kb of sequences upstream of selected target genes, an approach that has worked well in animals and plants with relatively small genomes. As presented earlier, however, this approach was often unsuccessful in Parhyale: from tens of reporters tested, only four robust native drivers had so far been identified (refs). The present work adds 5 new drivers to that collection, including ones with ubiquitous, neuron- and muscle-specific activities. This result comes from combining information on genome-wide chromatin accessibility and evolutionary conservation profiles.

We cannot at this point distinguish the relative contributions of chromatin profiling and sequence conservation to CRE discovery, because these sources of information were not tested separately. At minimum, we can state that (1) ATAC-seq profiles serve to identify robustly the promoters of candidate genes that are active in a particular cellular context (cell type and stage) and (2) coupling this information with sequence conservation narrows down candidate promoters and distant CREs by a factor of 4 to 10, since only a fraction of ATAC-seq peaks show sequence conservation (Figure 3D). This represents a great improvement in our ability to select candidates to test by transgenesis, the most labour-intensive step in the process."

Further, we have added this text to explain the advantages and cost savings of our low coverage sequencing strategy: "Our strategy of mapping regions of sequence conservation by direct mapping of short sequence reads across species is much more accessible than conventional strategies that rely on genome assembly. The latter require much higher sequence coverage (> 50x) from multiple libraries, long-read sequencing or other scaffolding methods, and complex bioinformatic pipelines to assemble large genomes. Moreover, these approaches are often compromised by high levels of polymorphism found in natural populations. We estimate that our approach is 5- to 10-fold cheaper than assembly-based methods, even excluding labor costs."

(5) Improve figure readability:

The authors could improve figure readability by introducing a schematic representation of the specimens and more references in Figures 4, 5 and Supplementary Figure 5. A schematic representation would help non-experts in Parhyale understand what they are looking at. Some figures might also benefit from improved color contrast (e.g., Figure 3 has very similar orange/red colors; black dots on a dark grey background are hard to distinguish).

We have added additional labels and explanations in the legends of Figures 4, 5 and Supplementary Figure 5, which we think will make the images more intelligible to the readers. In Figure 3 we modified the colouring in panels B and C to improve contrast.

(6) Quantify reporter validation efficiencies:

The authors should add a summary table/plot (e.g. n surviving, n fluorescent) or label the figures (n of specimens showing that pattern/n of specimens that do not show the pattern) with exact numbers to explicitly illustrate the observation. For example, Supplementary Table 3 contains excellent data for the putative developmental CREs tested, but the main text lacks equivalent quantification for successful reporters.

This information is already provided in Table 3.

(7) Discuss the rapid evolution of developmental CREs:

The failure to validate developmental CREs using conserved candidates may also reflect the rapid evolution and turnover of developmental enhancers, which can erode detectable sequence conservation over these phylogenetic distances. As a result, functionally relevant elements may have been excluded during candidate selection. It may be worth discussing this possibility alongside the proposed long-range and shadow enhancers hypothesis.

We added the phrase “or the rapid evolution of these enhancers leading to low sequence conservation" in the relevant part of the Discussion.

**Reviewer #2 (Public review):**

The manuscript by Forbes, Skafida, Karapidaki et al. concerns the in silico identification of cis-regulatory elements (CREs) in large genomes using chromatin accessibility (ATAC-seq) and sequence conservation (genomic DNA sequencing) data. They exemplify this method by applying it to identify novel CREs in Parhyale hawaiensis, which they validated using reporter constructs.

The results are convincing and are well supported by the data and validations. Identified CREs are valuable for researchers interested in the regulation of the expression of genes they control.

The methodology on the whole is also valid, as suggested by the results and previous publications on various taxa. Sequence conservation, as stated by the authors, was long used as a method to identify regions of non-coding DNA with functional and evolutionary constraints. The same applies to ATAC-seq data, which has also been used as a proxy for functional regions in different animals such as sea urchins and amphioxus. The methodology proposed is likely to be successfully used by researchers working on a variety of experimental organisms.

The authors do not use existing genome assemblies and use short-read sequencing to identify conserved regions, and while it is not conceptually novel, such an approach is becoming more and more viable and useful considering the recent advances in next-generation sequencing technology and the decrease in price of short-read sequencing.

We thank the reviewer for their comments and valuable feedback.

Two major weaknesses are:

(1) The novelty of the approach and its advantages should be more explicitly stated.

(2) The authors do not discuss in depth the strength of using a combination of two methods rather than either of the two, especially considering that previously known CREs do not overlap with conserved sequences.

We have added two paragraphs at the start of the Discussion to address the reviewer's comments 1 and 2 more explicitly (see response to reviewer 1, comment 4).

Previously known CREs do include some conserved sequences, see Suppl. Figure 7.

**Reviewer #2 (Recommendations for the authors):**

In addition to addressing the two above-mentioned weaknesses, the authors should address the following minor issues:

(1) It is difficult to refer to particular regions of text without line numbers.

Spelling of ChIPseq is inconsistent in the Introduction.

Spelling corrected. (Sorry for not including line numbering, we'll try to remember next time.)

(2) "6.4% of reads from P. aquilina, 4.1% of reads from P. darvishi, and 0.46 % of reads from P. plumicornis could be mapped unambiguously to the P. hawaiensis genome" seems quite low for closely related species. Do the authors expect such low rates?

Neutral nucleotide substitution rates in multicellular animals are in the order of 1 per site per 100 million years (e.g. https://pubmed.ncbi.nlm.nih.gov/12949132/) or a little lower (e.g. https://pubmed.ncbi.nlm.nih.gov/11792858/, https://pubmed.ncbi.nlm.nih.gov/34049492/). With the evolutionary times separating *P. hawaiensis* from *P. aquilina/darvishi* and *P. plumicornis* estimated at roughly 50 and 180 million years (2x25 and 2x90 million years, respectively), we expect a large fraction of neutrally evolving nucleotides in these genomes to have changed. We performed the read mapping using bowtie2, which requires a ~20 nt long perfect match with the reference sequence. We were therefore not surprised to obtain such low rates of read mapping. In fact, these low mapping rates (long divergence times) are important for islands of sequence conservation to stand out.

(3) "Of these, 37% are found in introns, 54% in intergenic regions, and 1% overlap with promoters (TSS), marking regions that evolve at a lower rate than surrounding non-coding sequences". The authors explain in the Methods why they omit exons, but in the Results and Discussion, it is not stated. In addition, discussing the conservation with exons would be helpful, and the % in exons should be compared to non-coding regions.

We have added "Of these, 8% are found in exons, likely reflecting conservation in protein-coding sequences".

(4) "Two of the 7 reporters we tested, named neuro5 and neuro6," if I understood correctly, neuro5 and neuro6 are CREs, however, they are named quite ambiguously, and their names can be mistaken for gene names.

Indeed, neuro5 and neur6 are the names of the CRE reporters. We have now added the names of the corresponding genes ("carrying CREs associated with the genes αTub and Cdk5α, respectively"). The gene names are also given in Table 3.

(5) Why was single-end sequencing done for E24?

We now explain this in the Methods: "Sequencing was carried out on an Illumina NextSeq 500 sequencer; we carried out single-end 76 bp sequencing for the first sample we generated (E24), and then switched to paired-end 76 bp sequencing for the other samples, because this leads to more specific read mapping."

(6) Syntax related to in-line references should be double checked as the following sentences are broken by parentheses, e.g., "updated in (Almazán et al. 2022)".

Corrected.

(7) Could the authors discuss the P. aquilina genome size, which was estimated to be 3-times less than P. hawaiensis? Considering that in their phylogeny these two species are closest, it is quite surprising that they have such differing genome sizes. Do you expect it to be true? If yes, what could be the reason?

As we explain in the manuscript, our estimates of genome size were obtained by dividing the total number of nucleotides sequenced by the estimated genome coverage, for each species. This method could overestimate genome sizes if there was a significant fraction of contaminating DNA in the preps, or a high degree of sequence variation that would prevent efficient mapping to BUSCO genes (both would underestimate genome coverage), but we find no evidence of this when we estimate the genome size of *P. hawaiensis* (see manuscript). We used the same method to estimate genome size in all four *Parhyale* species and have no reason to think that the method would be biased in one species and not in others. We therefore think that we have comparable estimates of genome size for the 4 species and the size difference is real.

Variations in genome size can be driven by changes in the fraction of repetitive sequences found in a genome. We therefore checked the proportion of repetitive elements in each *Parhyale* genome using dnaPipeTE (https://github.com/clemgoub/dnaPipeTE). Based on this method (which likely underestimates the repetitive genome content) we find that the genomes of *P. hawaiensis*, *P. aquiline*, *P. darvishi* and *P. plumicornis* contain 31%, 22%, 18% and 39% of repetitive sequences, respectively. These figures do not fully account for the differences in genome size (particularly since *P. darvishi* appears to have even fewer repetitive sequences than *P. aquiline*). We therefore hesitate to add this very preliminary analysis to the manuscript.

Of note, such rapid change in genome size is not unprecedented: in fruit flies genome size can vary more than 3-fold in species that have diverged over about 30 million years (https://elifesciences.org/articles/66405).

(8) Wording "and found a genome coverage of 5.8x, corresponding to a genome size of 3.0 Gbp instead of 3.6 Gbp" is confusing and unclear as to what the authors exactly did here.

We modified the sentence: "As a control, we followed the same procedure for *P. hawaiensis*, for which genome size is known (ref), and found a genome size of 3.0 Gbp instead of 3.6 Gbp (with a genome coverage of 5.8x)."

(9) In the figures and supplementary figures, the genome browser screenshots should also include tracks of macs2 called peaks (those in narrowPeak format).

Each ATACseq and sequence conservation track has its own set of peaks; we think that adding more tracks would overcrowd the figures. All the tracks (including called peaks) are provided as genome-browser-readable files in Supplementary Data files 1-3, so readers should be able to explore the data and reconstruct the figure panels without much effort.

**Reviewer #3 (Public review):**

Summary:

Forbes et al. present a new approach for identifying cis-regulatory elements in large genomes. Using Parhyale hawaiensis, a crustacean with a large genome (~3.6 Gb, comparable in size to the human genome), the authors show that current methods for identifying cis-regulatory elements, effective in smaller genomes, are markedly inefficient in organisms with large genomes. To address this limitation, they combine bulk ATAC-seq and single-cell (sc) ATAC-seq to identify chromatin regions that are either ubiquitously accessible or specifically accessible in particular cell types. They further integrate comparative genomics across multiple Parhyale species (P. hawaiensis, P. aquilina, and P. darvishi), selected at appropriate phylogenetic distances (20-95 million years divergence), to pinpoint conserved open chromatin regions likely under functional constraint.

Using this strategy, the authors predict a set of ubiquitous and cell-type-specific cis-regulatory elements. Importantly, they validate these predictions using rigorous transgenic reporter assays, convincingly demonstrating that their approach can successfully identify functional regulatory elements where previous methods had failed.

Strengths:

The approach introduced by Forbes et al. is conceptually straightforward, efficient, and readily transferable to other organisms. The validation experiments show not only that a substantial proportion of the predicted elements are functional, but also that the method is capable of identifying both ubiquitous and cell-type-specific regulatory elements. Given that the identification of regulatory regions remains a major bottleneck in understanding the molecular mechanisms underlying processes of development and regeneration, this work has the potential to make a significant impact in developmental and regeneration biology, particularly for studies involving non-model organisms with large genomes.

An additional strength is the demonstration that only the genome of the focal species requires high-quality sequencing and assembly. In contrast, species used solely for comparative analysis can be sequenced at low coverage without assembly, substantially reducing costs and increasing the accessibility of the approach.

Weaknesses:

While the method is effective in identifying regulatory elements that are active ubiquitously or in differentiated cell types, it failed in detecting elements associated with developmentally regulated genes. This may be due to trivial reasons, such as a very low level of expression of the selected genes. However, as acknowledged by the authors, it may also indicate inherent challenges in identifying regulatory elements associated with developmentally dynamic gene regulation, compared to those associated with genes expressed in differentiated cell types.

A second limitation, also acknowledged by the authors, is the absence of chromatin conformation capture data, which would help link distal regulatory elements to their target genes. This limitation may be particularly relevant for developmentally regulated genes, where long-range regulatory interactions may be critical.

Addressing these limitations will be an important direction for future work. Nonetheless, the approach as presented in this manuscript represents a key contribution that sets the stage for further methodological advances in the identification of cis-regulatory elements in large genomes.

**Reviewer #3 (Recommendations for the authors):**

I have no specific comment for the authors. While in my opinion the study has two limitations (as described in the public review), these are clearly acknowledged and properly discussed in the manuscript.

The manuscript is extremely well written. It has been a great pleasure to read it. Excellent job!

Thank you!
